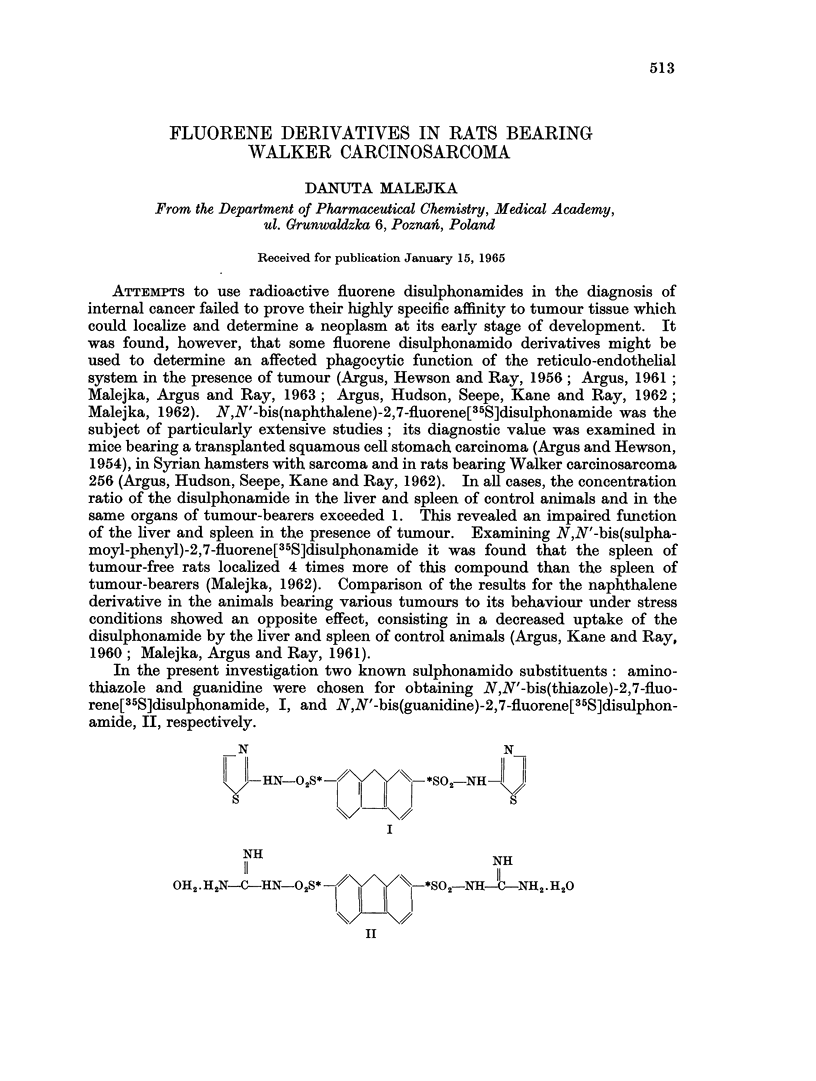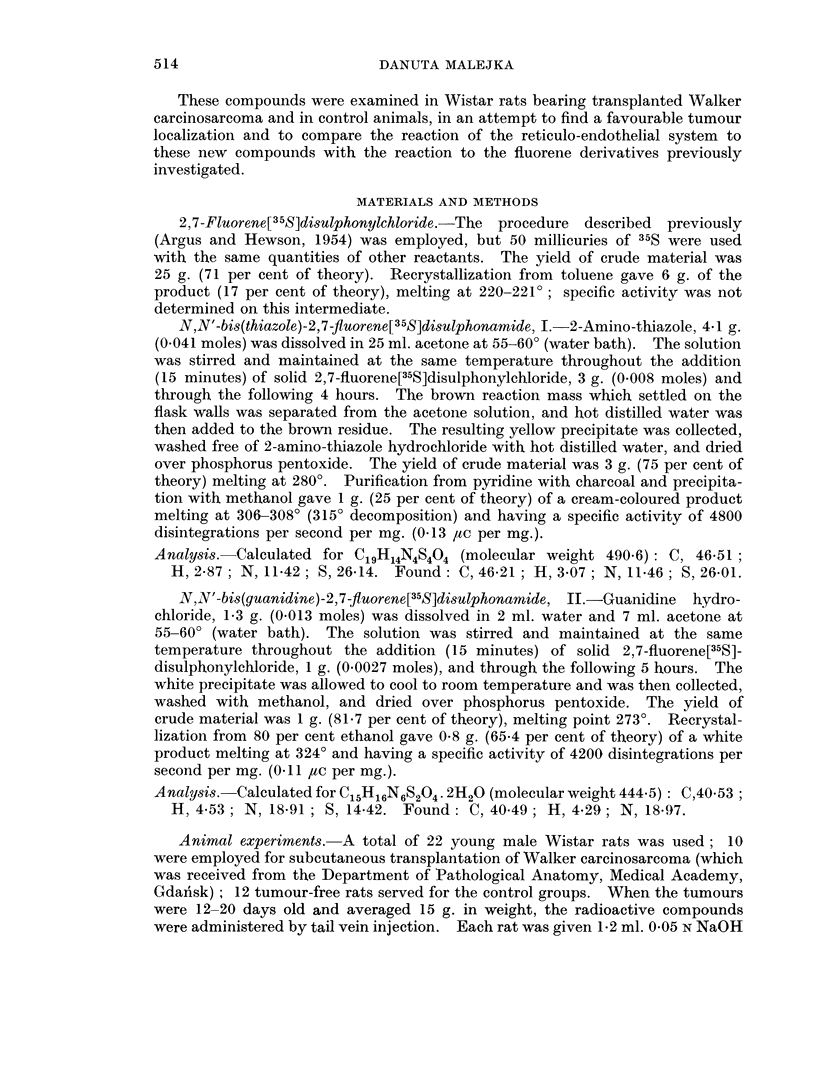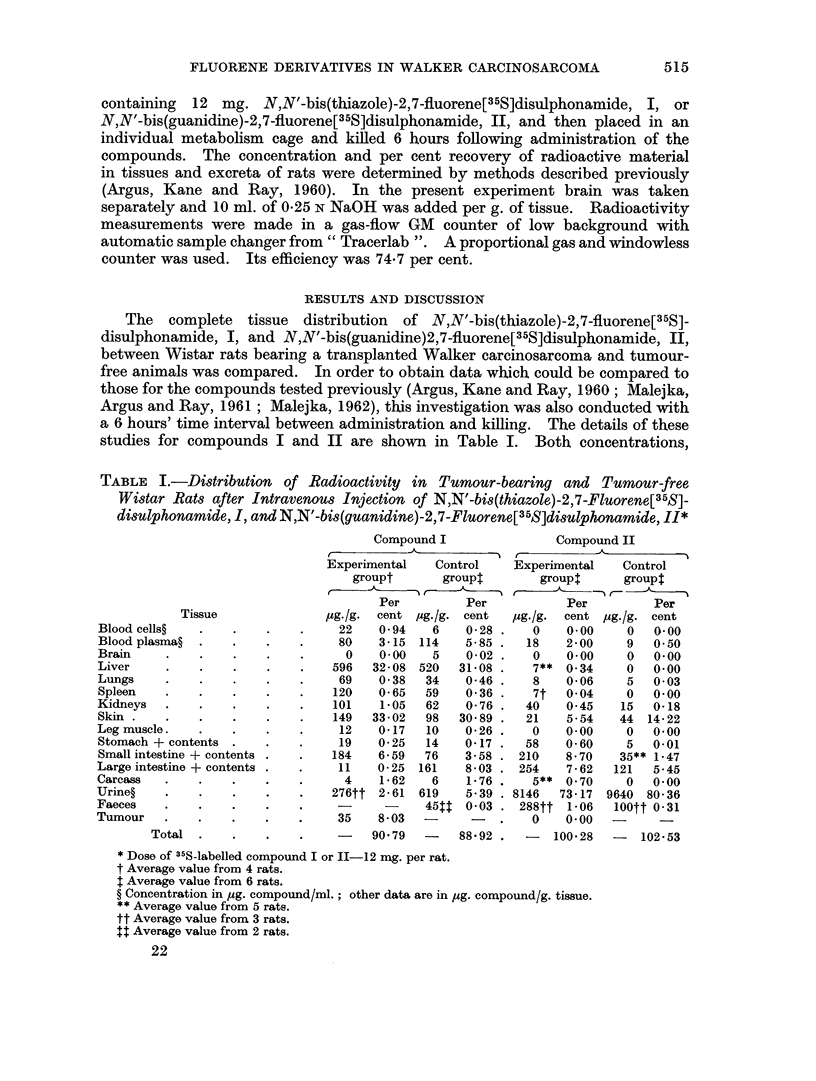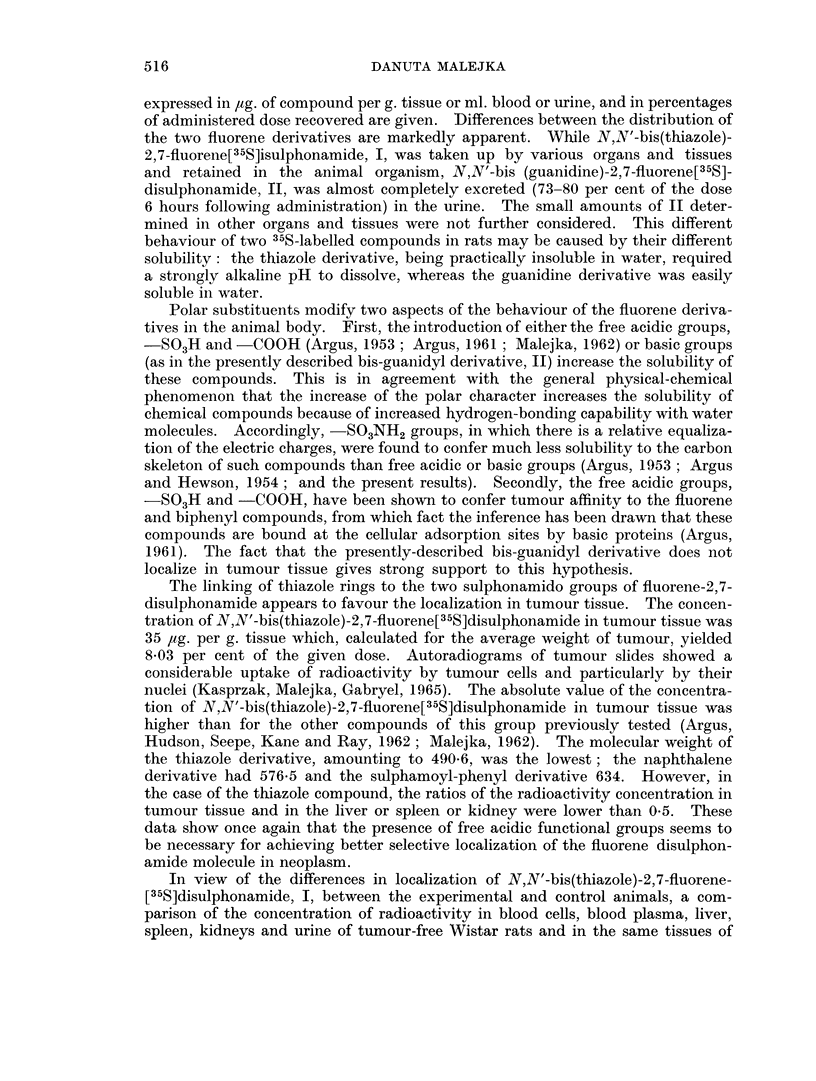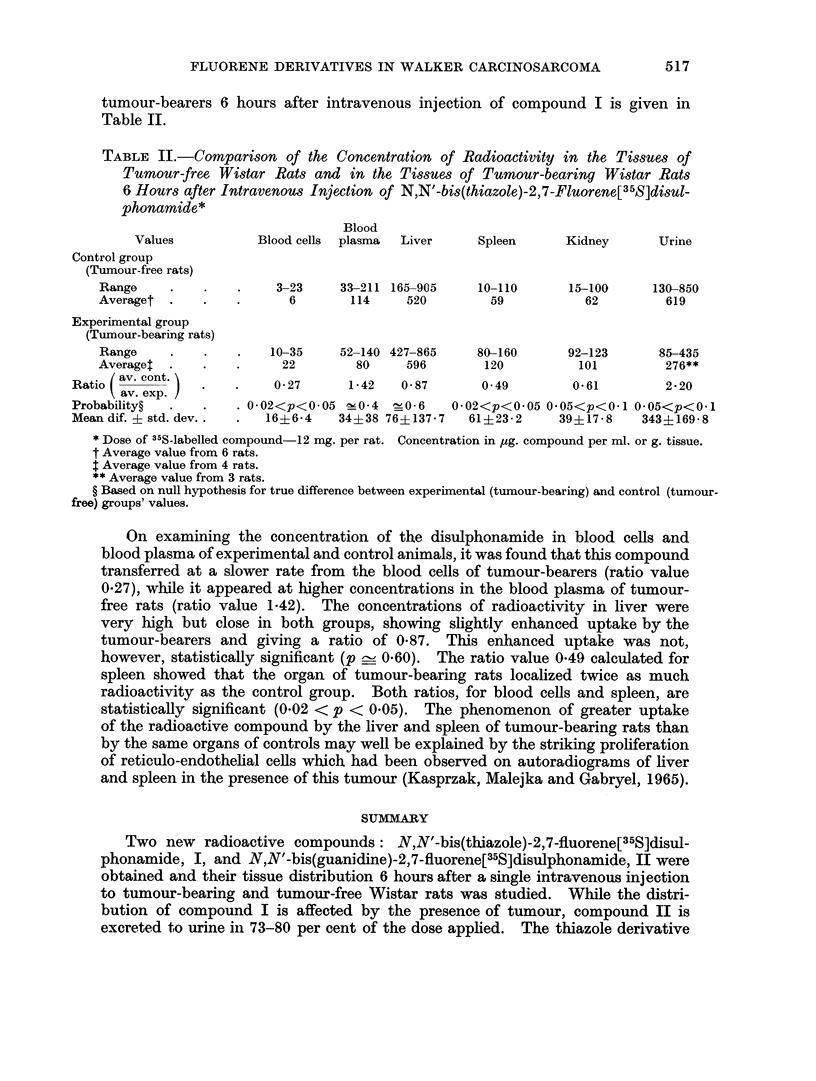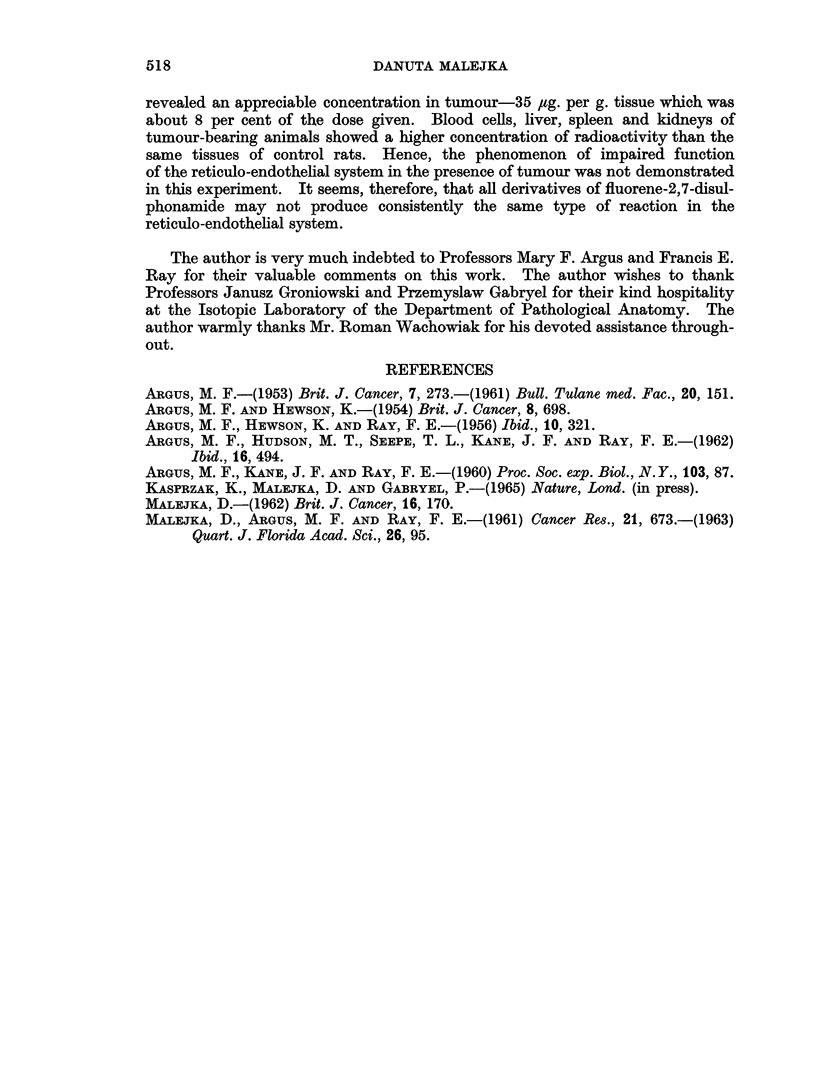# Fluorene derivatives in rats bearing Walker carcinosarcoma.

**DOI:** 10.1038/bjc.1965.62

**Published:** 1965-09

**Authors:** D. Malejka


### FLUORENE DERIVATIVES IN RATS BEARING WALKER CARCINOSARCOMA

DANUTA MALEJKA

*From the Department of Pharmaceutical Chemistry, Medical Academy, ul. Grunwaldzka* 6, *Poznań, Poland*

Received for publication January 15, 1965

ATTEMPTS to use radioactive fluorene disulphonamides in the diagnosis of internal cancer failed to prove their highly specific affinity to tumour tissue which could localize and determine a neoplasm at its early stage of development. It was found, however, that some fluorene disulphonamido derivatives might be used to determine an affected phagocytic function of the reticulo-endothelial system in the presence of tumour (Argus, Hewson and Ray, 1956 ; Argus, 1961 ; Malejka, Argus and Ray, 1963 ; Argus, Hudson, Seepe, Kane and Ray, 1962 ; Malejka, 1962). *N*,*N*′-bis(naphthalene)-2,7-fluorene[$^{35}$S]disulphonamide was the subject of particularly extensive studies ; its diagnostic value was examined in mice bearing a transplanted squamous cell stomach carcinoma (Argus and Hewson, 1954), in Syrian hamsters with sarcoma and in rats bearing Walker carcinosarcoma 256 (Argus, Hudson, Seepe, Kane and Ray, 1962). In all cases, the concentration ratio of the disulphonamide in the liver and spleen of control animals and in the same organs of tumour-bearers exceeded 1. This revealed an impaired function of the liver and spleen in the presence of tumour. Examining *N*,*N*′-bis(sulphamoyl-phenyl)-2,7-fluorene[$^{35}$S]disulphonamide it was found that the spleen of tumour-free rats localized 4 times more of this compound than the spleen of tumour-bearers (Malejka, 1962). Comparison of the results for the naphthalene derivative in the animals bearing various tumours to its behaviour under stress conditions showed an opposite effect, consisting in a decreased uptake of the disulphonamide by the liver and spleen of control animals (Argus, Kane and Ray, 1960 ; Malejka, Argus and Ray, 1961).

In the present investigation two known sulphonamido substituents : aminothiazole and guanidine were chosen for obtaining *N*,*N*′-bis(thiazole)-2,7-fluorene[$^{35}$S]disulphonamide, I, and *N*,*N*′-bis(guanidine)-2,7-fluorene[$^{35}$S]disulphonamide, II, respectively.

I

II

These compounds were examined in Wistar rats bearing transplanted Walker carcinosarcoma and in control animals, in an attempt to find a favourable tumour localization and to compare the reaction of the reticulo-endothelial system to these new compounds with the reaction to the fluorene derivatives previously investigated.

### MATERIALS AND METHODS

*2,7-Fluorene[$^{35}$S]disulphonylchloride.*—The procedure described previously (Argus and Hewson, 1954) was employed, but 50 millicuries of $^{35}$S were used with the same quantities of other reactants. The yield of crude material was 25 g. (71 per cent of theory). Recrystallization from toluene gave 6 g. of the product (17 per cent of theory), melting at 220–221° ; specific activity was not determined on this intermediate.

*N,N′-bis(thiazole)-2,7-fluorene[$^{35}$S]disulphonamide,* I.—2-Amino-thiazole, 4·1 g. (0·041 moles) was dissolved in 25 ml. acetone at 55–60° (water bath). The solution was stirred and maintained at the same temperature throughout the addition (15 minutes) of solid 2,7-fluorene[$^{35}$S]disulphonylchloride, 3 g. (0·008 moles) and through the following 4 hours. The brown reaction mass which settled on the flask walls was separated from the acetone solution, and hot distilled water was then added to the brown residue. The resulting yellow precipitate was collected, washed free of 2-amino-thiazole hydrochloride with hot distilled water, and dried over phosphorus pentoxide. The yield of crude material was 3 g. (75 per cent of theory) melting at 280°. Purification from pyridine with charcoal and precipitation with methanol gave 1 g. (25 per cent of theory) of a cream-coloured product melting at 306–308° (315° decomposition) and having a specific activity of 4800 disintegrations per second per mg. (0·13 $\mu$c per mg.).

*Analysis.*—Calculated for $C_{19}H_{14}N_4S_4O_4$ (molecular weight 490·6) : C, 46·51 ; H, 2·87 ; N, 11·42 ; S, 26·14. Found : C, 46·21 ; H, 3·07 ; N, 11·46 ; S, 26·01.

*N,N′-bis(guanidine)-2,7-fluorene[$^{35}$S]disulphonamide,* II.—Guanidine hydrochloride, 1·3 g. (0·013 moles) was dissolved in 2 ml. water and 7 ml. acetone at 55–60° (water bath). The solution was stirred and maintained at the same temperature throughout the addition (15 minutes) of solid 2,7-fluorene[$^{35}$S]-disulphonylchloride, 1 g. (0·0027 moles), and through the following 5 hours. The white precipitate was allowed to cool to room temperature and was then collected, washed with methanol, and dried over phosphorus pentoxide. The yield of crude material was 1 g. (81·7 per cent of theory), melting point 273°. Recrystallization from 80 per cent ethanol gave 0·8 g. (65·4 per cent of theory) of a white product melting at 324° and having a specific activity of 4200 disintegrations per second per mg. (0·11 $\mu$c per mg.).

*Analysis.*—Calculated for $C_{15}H_{16}N_6S_2O_4 . 2H_2O$ (molecular weight 444·5) : C,40·53 ; H, 4·53 ; N, 18·91 ; S, 14·42. Found : C, 40·49 ; H, 4·29 ; N, 18·97.

*Animal experiments.*—A total of 22 young male Wistar rats was used ; 10 were employed for subcutaneous transplantation of Walker carcinosarcoma (which was received from the Department of Pathological Anatomy, Medical Academy, Gdańsk) ; 12 tumour-free rats served for the control groups. When the tumours were 12–20 days old and averaged 15 g. in weight, the radioactive compounds were administered by tail vein injection. Each rat was given 1·2 ml. 0·05 N NaOH

containing 12 mg. *N,N'*-bis(thiazole)-2,7-fluorene[$^{35}$S]disulphonamide, I, or *N,N'*-bis(guanidine)-2,7-fluorene[$^{35}$S]disulphonamide, II, and then placed in an individual metabolism cage and killed 6 hours following administration of the compounds. The concentration and per cent recovery of radioactive material in tissues and excreta of rats were determined by methods described previously (Argus, Kane and Ray, 1960). In the present experiment brain was taken separately and 10 ml. of 0·25 N NaOH was added per g. of tissue. Radioactivity measurements were made in a gas-flow GM counter of low background with automatic sample changer from " Tracerlab ". A proportional gas and windowless counter was used. Its efficiency was 74·7 per cent.

### RESULTS AND DISCUSSION

The complete tissue distribution of *N,N'*-bis(thiazole)-2,7-fluorene[$^{35}$S]-disulphonamide, I, and *N,N'*-bis(guanidine)2,7-fluorene[$^{35}$S]disulphonamide, II, between Wistar rats bearing a transplanted Walker carcinosarcoma and tumour-free animals was compared. In order to obtain data which could be compared to those for the compounds tested previously (Argus, Kane and Ray, 1960 ; Malejka, Argus and Ray, 1961 ; Malejka, 1962), this investigation was also conducted with a 6 hours' time interval between administration and killing. The details of these studies for compounds I and II are shown in Table I. Both concentrations,

TABLE I.—*Distribution of Radioactivity in Tumour-bearing and Tumour-free Wistar Rats after Intravenous Injection of* N,N'-*bis(thiazole)-2,7-Fluorene*[$^{35}$*S*]-*disulphonamide, I, and* N,N'-*bis(guanidine)-2,7-Fluorene*[$^{35}$*S*]*disulphonamide, II**

| | Compound I | | | | Compound II | | | |
|---|---|---|---|---|---|---|---|---|
| | Experimental group† | | Control group‡ | | Experimental group‡ | | Control group‡ | |
| Tissue | µg./g. | Per cent | µg./g. | Per cent | µg./g. | Per cent | µg./g. | Per cent |
| Blood cells§ | 22 | 0·94 | 6 | 0·28 | 0 | 0·00 | 0 | 0·00 |
| Blood plasma§ | 80 | 3·15 | 114 | 5·85 | 18 | 2·00 | 9 | 0·50 |
| Brain | 0 | 0·00 | 5 | 0·02 | 0 | 0·00 | 0 | 0·00 |
| Liver | 596 | 32·08 | 520 | 31·08 | 7** | 0·34 | 0 | 0·00 |
| Lungs | 69 | 0·38 | 34 | 0·46 | 8 | 0·06 | 5 | 0·03 |
| Spleen | 120 | 0·65 | 59 | 0·36 | 7† | 0·04 | 0 | 0·00 |
| Kidneys | 101 | 1·05 | 62 | 0·76 | 40 | 0·45 | 15 | 0·18 |
| Skin | 149 | 33·02 | 98 | 30·89 | 21 | 5·54 | 44 | 14·22 |
| Leg muscle | 12 | 0·17 | 10 | 0·26 | 0 | 0·00 | 0 | 0·00 |
| Stomach + contents | 19 | 0·25 | 14 | 0·17 | 58 | 0·60 | 5 | 0·01 |
| Small intestine + contents | 184 | 6·59 | 76 | 3·58 | 210 | 8·70 | 35** | 1·47 |
| Large intestine + contents | 11 | 0·25 | 161 | 8·03 | 254 | 7·62 | 121 | 5·45 |
| Carcass | 4 | 1·62 | 6 | 1·76 | 5** | 0·70 | 0 | 0·00 |
| Urine§ | 276†† | 2·61 | 619 | 5·39 | 8146 | 73·17 | 9640 | 80·36 |
| Faeces | — | — | 45‡‡ | 0·03 | 288†† | 1·06 | 100†† | 0·31 |
| Tumour | 35 | 8·03 | — | — | 0 | 0·00 | — | — |
| Total | — | 90·79 | — | 88·92 | — | 100·28 | — | 102·53 |

* Dose of $^{35}$S-labelled compound I or II—12 mg. per rat.
† Average value from 4 rats.
‡ Average value from 6 rats.
§ Concentration in µg. compound/ml. ; other data are in µg. compound/g. tissue.
** Average value from 5 rats.
†† Average value from 3 rats.
‡‡ Average value from 2 rats.

expressed in $\mu$g. of compound per g. tissue or ml. blood or urine, and in percentages of administered dose recovered are given. Differences between the distribution of the two fluorene derivatives are markedly apparent. While *N*,*N'*-bis(thiazole)-2,7-fluorene[$^{35}$S]isulphonamide, I, was taken up by various organs and tissues and retained in the animal organism, *N*,*N'*-bis (guanidine)-2,7-fluorene[$^{35}$S]-disulphonamide, II, was almost completely excreted (73–80 per cent of the dose 6 hours following administration) in the urine. The small amounts of II determined in other organs and tissues were not further considered. This different behaviour of two $^{35}$S-labelled compounds in rats may be caused by their different solubility : the thiazole derivative, being practically insoluble in water, required a strongly alkaline pH to dissolve, whereas the guanidine derivative was easily soluble in water.

Polar substituents modify two aspects of the behaviour of the fluorene derivatives in the animal body. First, the introduction of either the free acidic groups, —$SO_3H$ and —COOH (Argus, 1953 ; Argus, 1961 ; Malejka, 1962) or basic groups (as in the presently described bis-guanidyl derivative, II) increase the solubility of these compounds. This is in agreement with the general physical-chemical phenomenon that the increase of the polar character increases the solubility of chemical compounds because of increased hydrogen-bonding capability with water molecules. Accordingly, —$SO_3NH_2$ groups, in which there is a relative equalization of the electric charges, were found to confer much less solubility to the carbon skeleton of such compounds than free acidic or basic groups (Argus, 1953 ; Argus and Hewson, 1954 ; and the present results). Secondly, the free acidic groups, —$SO_3H$ and —COOH, have been shown to confer tumour affinity to the fluorene and biphenyl compounds, from which fact the inference has been drawn that these compounds are bound at the cellular adsorption sites by basic proteins (Argus, 1961). The fact that the presently-described bis-guanidyl derivative does not localize in tumour tissue gives strong support to this hypothesis.

The linking of thiazole rings to the two sulphonamido groups of fluorene-2,7-disulphonamide appears to favour the localization in tumour tissue. The concentration of *N*,*N'*-bis(thiazole)-2,7-fluorene[$^{35}$S]disulphonamide in tumour tissue was 35 $\mu$g. per g. tissue which, calculated for the average weight of tumour, yielded 8·03 per cent of the given dose. Autoradiograms of tumour slides showed a considerable uptake of radioactivity by tumour cells and particularly by their nuclei (Kasprzak, Malejka, Gabryel, 1965). The absolute value of the concentration of *N*,*N'*-bis(thiazole)-2,7-fluorene[$^{35}$S]disulphonamide in tumour tissue was higher than for the other compounds of this group previously tested (Argus, Hudson, Seepe, Kane and Ray, 1962 ; Malejka, 1962). The molecular weight of the thiazole derivative, amounting to 490·6, was the lowest ; the naphthalene derivative had 576·5 and the sulphamoyl-phenyl derivative 634. However, in the case of the thiazole compound, the ratios of the radioactivity concentration in tumour tissue and in the liver or spleen or kidney were lower than 0·5. These data show once again that the presence of free acidic functional groups seems to be necessary for achieving better selective localization of the fluorene disulphonamide molecule in neoplasm.

In view of the differences in localization of *N*,*N'*-bis(thiazole)-2,7-fluorene-[$^{35}$S]disulphonamide, I, between the experimental and control animals, a comparison of the concentration of radioactivity in blood cells, blood plasma, liver, spleen, kidneys and urine of tumour-free Wistar rats and in the same tissues of tumour-bearers 6 hours after intravenous injection of compound I is given in Table II.

TABLE II.—*Comparison of the Concentration of Radioactivity in the Tissues of Tumour-free Wistar Rats and in the Tissues of Tumour-bearing Wistar Rats 6 Hours after Intravenous Injection of* N,N'-*bis*(*thiazole*)-2,7-*Fluorene*[$^{35}S$]*disulphonamide**

| Values | Blood cells | Blood plasma | Liver | Spleen | Kidney | Urine |
|---|---|---|---|---|---|---|
| Control group (Tumour-free rats) | | | | | | |
| Range | 3–23 | 33–211 | 165–905 | 10–110 | 15–100 | 130–850 |
| Average† | 6 | 114 | 520 | 59 | 62 | 619 |
| Experimental group (Tumour-bearing rats) | | | | | | |
| Range | 10–35 | 52–140 | 427–865 | 80–160 | 92–123 | 85–435 |
| Average‡ | 22 | 80 | 596 | 120 | 101 | 276** |
| Ratio $\left(\frac{\text{av. cont.}}{\text{av. exp.}}\right)$ | 0·27 | 1·42 | 0·87 | 0·49 | 0·61 | 2·20 |
| Probability§ | $0·02<p<0·05$ | $\cong 0·4$ | $\cong 0·6$ | $0·02<p<0·05$ | $0·05<p<0·1$ | $0·05<p<0·1$ |
| Mean dif. ± std. dev. | 16±6·4 | 34±38 | 76±137·7 | 61±23·2 | 39±17·8 | 343±169·8 |

* Dose of $^{35}$S-labelled compound—12 mg. per rat. Concentration in $\mu$g. compound per ml. or g. tissue.
† Average value from 6 rats.
‡ Average value from 4 rats.
** Average value from 3 rats.
§ Based on null hypothesis for true difference between experimental (tumour-bearing) and control (tumour-free) groups' values.

On examining the concentration of the disulphonamide in blood cells and blood plasma of experimental and control animals, it was found that this compound transferred at a slower rate from the blood cells of tumour-bearers (ratio value 0·27), while it appeared at higher concentrations in the blood plasma of tumour-free rats (ratio value 1·42). The concentrations of radioactivity in liver were very high but close in both groups, showing slightly enhanced uptake by the tumour-bearers and giving a ratio of 0·87. This enhanced uptake was not, however, statistically significant ($p \cong 0·60$). The ratio value 0·49 calculated for spleen showed that the organ of tumour-bearing rats localized twice as much radioactivity as the control group. Both ratios, for blood cells and spleen, are statistically significant ($0·02 < p < 0·05$). The phenomenon of greater uptake of the radioactive compound by the liver and spleen of tumour-bearing rats than by the same organs of controls may well be explained by the striking proliferation of reticulo-endothelial cells which had been observed on autoradiograms of liver and spleen in the presence of this tumour (Kasprzak, Malejka and Gabryel, 1965).

### SUMMARY

Two new radioactive compounds: *N*,*N'*-bis(thiazole)-2,7-fluorene[$^{35}$S]disulphonamide, I, and *N*,*N'*-bis(guanidine)-2,7-fluorene[$^{35}$S]disulphonamide, II were obtained and their tissue distribution 6 hours after a single intravenous injection to tumour-bearing and tumour-free Wistar rats was studied. While the distribution of compound I is affected by the presence of tumour, compound II is excreted to urine in 73–80 per cent of the dose applied. The thiazole derivative revealed an appreciable concentration in tumour—35 $\mu$g. per g. tissue which was about 8 per cent of the dose given. Blood cells, liver, spleen and kidneys of tumour-bearing animals showed a higher concentration of radioactivity than the same tissues of control rats. Hence, the phenomenon of impaired function of the reticulo-endothelial system in the presence of tumour was not demonstrated in this experiment. It seems, therefore, that all derivatives of fluorene-2,7-disulphonamide may not produce consistently the same type of reaction in the reticulo-endothelial system.

The author is very much indebted to Professors Mary F. Argus and Francis E. Ray for their valuable comments on this work. The author wishes to thank Professors Janusz Groniowski and Przemyslaw Gabryel for their kind hospitality at the Isotopic Laboratory of the Department of Pathological Anatomy. The author warmly thanks Mr. Roman Wachowiak for his devoted assistance throughout.

### REFERENCES

Argus, M. F.—(1953) *Brit. J. Cancer*, **7**, 273.—(1961) *Bull. Tulane med. Fac.*, **20**, 151.
Argus, M. F. and Hewson, K.—(1954) *Brit. J. Cancer*, **8**, 698.
Argus, M. F., Hewson, K. and Ray, F. E.—(1956) *Ibid.*, **10**, 321.
Argus, M. F., Hudson, M. T., Seepe, T. L., Kane, J. F. and Ray, F. E.—(1962) *Ibid.*, **16**, 494.
Argus, M. F., Kane, J. F. and Ray, F. E.—(1960) *Proc. Soc. exp. Biol., N.Y.*, **103**, 87.
Kasprzak, K., Malejka, D. and Gabryel, P.—(1965) *Nature, Lond.* (in press).
Malejka, D.—(1962) *Brit. J. Cancer*, **16**, 170.
Malejka, D., Argus, M. F. and Ray, F. E.—(1961) *Cancer Res.*, **21**, 673.—(1963) *Quart. J. Florida Acad. Sci.*, **26**, 95.